# The Sol Genomics Network (SGN)—from genotype to phenotype to breeding

**DOI:** 10.1093/nar/gku1195

**Published:** 2014-11-26

**Authors:** Noe Fernandez-Pozo, Naama Menda, Jeremy D. Edwards, Surya Saha, Isaak Y. Tecle, Susan R. Strickler, Aureliano Bombarely, Thomas Fisher-York, Anuradha Pujar, Hartmut Foerster, Aimin Yan, Lukas A. Mueller

**Affiliations:** 1 Boyce Thompson Institute for Plant Research, Ithaca, NY 14853, USA; 2 Dale Bumpers National Rice Research Center, Stuttgart, AR 72160, USA; 3 Department of Plant Pathology and Plant-Microbe Biology, Cornell University, Ithaca, NY 14853, USA; 4 Department of Horticulture, Virginia Polytechnic Institute and State University, Blacksburg, VA 24061–0002, USA; 5 Department of Plant Breeding, Cornell University, Ithaca, NY 14853, USA

## Abstract

The Sol Genomics Network (SGN, http://solgenomics.net) is a web portal with genomic and phenotypic data, and analysis tools for the Solanaceae family and close relatives. SGN hosts whole genome data for an increasing number of Solanaceae family members including tomato, potato, pepper, eggplant, tobacco and *Nicotiana benthamiana*. The database also stores loci and phenotype data, which researchers can upload and edit with user-friendly web interfaces. Tools such as BLAST, GBrowse and JBrowse for browsing genomes, expression and map data viewers, a locus community annotation system and a QTL analysis tools are available. A new tool was recently implemented to improve Virus-Induced Gene Silencing (VIGS) constructs called the SGN VIGS tool. With the growing genomic and phenotypic data in the database, SGN is now advancing to develop new web-based breeding tools and implement the code and database structure for other species or clade-specific databases.

## INTRODUCTION

In recent years, high-quality reference genomes have been generated for many plant genomes including *Arabidopsis*, rice, maize, poplar, grapevine and tomato. The Sol Genomics Network (SGN, http://solgenomics.net) was originally created to store genetic and genomic information, mainly genetic mapping and expressed sequence tags data for Solanaceous species, such as tomato, potato, pepper, eggplant, petunia and tobacco. SGN is the hub for the tomato genome sequencing project and hosts whole genome sequences of tomato wild accessions, potato, pepper, *Nicotiana benthamiana*, tobacco and eggplant. It also hosts non-Solanaceae reference genomes, such as *Arabidopsis thaliana* (1), *Vitis vinifera* (2) and rice (3–6) for comparative analysis.

However, reference genomes by themselves, although useful for addressing many questions, are of limited usefulness for understanding a key question of biology—how phenotypes result from genotypes. To address this question, also sometimes referred to as ‘G2P’, information beyond a single reference genome is needed. To address G2P questions, a genome database must not only store the reference genome information (such as gene models, expression data and repeat information), but also include two additional important data types: sequence variation data and phenotypic data. Based on these data, analyses such as Genome-Wide Association Studies are possible that can identify genomic regions contributing to a trait of interest if necessary criteria are met. Since genotyping has become cheaper to perform, it is the phenotyping that presents a bottleneck (7), therefore, new phenotyping systems are needed. A priority for the SGN database has been to provide a comprehensive module for storing genotypic and phenotypic information as databases that integrate genotypes and phenotypes seamlessly are required for breeding.

An important application of the growing genomic and phenotypic data is crop improvement. What roles can genome databases play in breeding better crops? Genomic data and breeding data need to be tightly integrated to inform breeding. Breeding data are a new dimension for genome databases, mainly because breeding is a complex process that entails crossing, planting and selecting plants, all of which should ideally be managed from within the genome database. The SGN platform has recently been supplemented with a breeding management system that is tailored to genomic-based breeding approaches, such as Genomic Selection (8). It is currently implemented in a database called Cassavabase (http://cassavabase.org/) that is based on the SGN platform.

## THE SGN GENOME DATABASE

Genome sequences form the backbone of the genomics approach and a significant component of the SGN database is devoted to the storage of genome information. The genome database implements functionality that is typical for model organism databases (MODs), such as genome viewers and a locus database, but there are some notable differences to other MODs. SGN is a community-driven database, in which data is under the direct control of users who can update or delete the information he or she submitted at any time through easy-to-use web interfaces. Such community-curated databases are still rare among MODs, most of which are being maintained exclusively by in-house curators. While community-based systems cannot replace professional curation, in-house curator-based models are much more expensive to operate and are not scalable for large research communities. The problem with community-based systems is the potential lack of community involvement and interest. Our experience is that community involvement requires active outreach and will not come about spontaneously. Currently, SGN has 132 active community curators, who are responsible for editing of 570 distinct loci, with a total of 661 edits and data contributions. The majority of these curators (>90%) were approached by an SGN curator (email or in person at conferences and meetings). Another feature of the SGN system is its support for multiple species, with the comparative viewer, an SGN custom application (9).

A well-curated genome provides a list of annotated loci and SGN has a comprehensive system for dealing with loci. Each locus has a locus detail page that contains all the information about it in the database. The page is divided into sections organized by data type. The first section, ‘Locus details’, contains basic information about a locus, such as its name, symbol and textual description. This section is editable only by a specially assigned user called the ‘locus editor’ and SGN-based curators. The locus editor is a scientist who is considered an expert on the locus, preferably someone involved in its characterization. The locus editor is assigned by an SGN curator after contacting authors of publications involving the gene. Users from the community can also request locus editor privileges through a link on the each locus page. SGN curators will then review the request and grant it if the user fulfills locus editor criteria, which are affiliation with research institute and proven work related to the gene. While only the locus editors can edit the gene name, symbol and function, other SGN users with ‘submitter’ privileges can contribute to all the other sections on the page, such as associated publications, GenBank sequences, images and ontology annotations. Each submitted item is owned by the respective submitter, who has the right to further edit or delete the item. A full description of the interface is presented in Menda *et al.* (6), with newly added features of linking the locus with a gene model, which provides cross-links between the genome and the experimentally described genetic locus and manual curation of gene families (10). The locus editing is also supported by an SGN custom tool called solQTL (11), which allows users to upload raw phenotype and genotype data from QTL studies to the SGN database. Users can use the tool to run QTL analysis for their traits and link predicted QTLs to annotated genomic regions in the database. This cross-linking enables them to exploit the rich genomic data annotations and perhaps identify candidate genes underlying the phenotype of their traits.

Interacting with users is a critical part of a web portal, such as SGN. We have implemented a comprehensive tracking system for managing user feedback and our internal software development processes. Users submit feedback and feature requests via the web contact form or email to sgn-feedback@solgenomics.net. We use the Trac ticket management system (http://trac.edgewall.org/) to track all user interactions. Each ticket is assigned to an SGN developer for follow-up and resolution in a timely manner. The source code for SGN and for all the custom tools developed by the SGN programmers is maintained publicly in the Github open source repository (https://github.com/solgenomics/sgn). A detailed description of the technology behind the SGN platform is available in previous publications of the SGN database (5). The progress of website feature requests by users as well as internal software development tasks is managed with the Github Issues system.

Since the last publication of SGN in the Nucleic Acids Research Database issue (5), 15 Solanaceae genome sequences and draft genomes have been published (12–20), including the closely related species *Coffea canephora* (21) (Table 1) while other genomes, such as *Petunia axillaris* and *Petunia inflata*, will be published soon (22). All the public data from these species, including different genome builds and their annotations are stored and shared on the SGN FTP site (ftp://ftp.solgenomics.net/genomes/). Annotations include the gene (transcripts and proteins) sequences and their coordinates in the genome, in FASTA and GFF formats, respectively. The information of the finished genomes, i.e. the ones assembled to the level of pseudo-molecules, is incorporated into the SGN database and tools. The draft genomes data are only added to the SGN tools including Basic Local Alignment Search Tool (BLAST) (23), genome browsers and SolCyc biochemical pathways, with some updates on-going or imminent. SGN BLAST, has a new interface to find sequences based on sequence similarity. Now it is easier to access the most popular BLAST databases from Solanaceae model plants. It includes genome data sets (chromosomes or scaffolds), gene and protein sequences, transcriptome contigs, markers, organelles and other data sets for Solanaceae species.

**Table 1.** List of published genome sequences and draft genomes from Solanaceae and close relative species

Published genome	Date of publication	Journal	Reference
Potato (*Solanum tuberosum*)	July 2011	Nature	(12)
Tomato (*Solanum lycopersicum* ‘Heinz 1706′)	May 2012	Nature	(13)
*Solanum pimpinellifolium* Draft	May 2012	Nature	(13)
*Nicotiana benthamiana* Draft	July 2012	MPMI	(14)
*Nicotiana sylvestris* Draft	June 2013	Genome Biol.	(15)
*Nicotiana tomentosiformis* Draft	June 2013	Genome Biol.	(15)
Pepper (*Capsicum annuum*) CM334	Jan. 2014	Nat. genet.	(16)
*Capsicum annuum Zunla-1*	Jan. 2014	PNAS	(17)
*Capsicum annuum glabriusculum*	Jan. 2014	PNAS	(17)
Tobacco (*Nicotiana tabacum*) TN90 Draft	May 2014	Nat. Commun.	(18)
*Nicotiana tabacum K326* Draft	May 2014	Nat. Commun.	(18)
*Nicotiana tabacum BX* Draft	May 2014	Nat. Commun.	(18)
Eggplant (*Solanum melongena*) Draft	Aug. 2014	DNA Res.	(19)
*Solanum pennellii* Draft	Sep. 2014	Nat. Genet.	(20)
Coffee (*Coffea canephora*)	Sep. 2014	Science	(21)

SGN also provides SolCyc, a set of Pathway/Genome Databases (PGDB) based on MetaCyc information that provide an encyclopedia of metabolic pathways and enzymatic reactions for the Solanaceae species (24). SolCyc was created using MetaCyc as a reference database and the Pathologic component of Pathway Tools software (25). The Metabolic networks were predicted based on the annotations of the genes from the Solanaceae species. Previous SolCyc PGDBs (5) were based on the annotations from contigs from transcriptome assemblies (unigenes). Current pathways were updated using the annotations from the gene models predicted for the reference genome sequence of tomato (LycoCyc), potato (PotatoCyc), *N. benthamiana* (BenthamianaCyc) and pepper (CapCyc). Other species will be updated soon.

Another important feature of genome databases are genome viewers. Genome viewers enable users to explore a genome or genomic region using an intuitive and graphical display of the genome. They also present the users with informative tracks like expression data, gene models, markers, SNPs and many other data that allows users to analyze all this information visually to facilitate the study of a genomic region or feature. SGN has provided this functionality in the past using the GBrowse system (26), but recently added the newer JBrowse (27), which offers a more modern and fluid user experience. All SGN genomes are being migrated to this new browser. As of October 2014, tomato genome version 2.50 and annotation ITAG2.4, tomato genome version 2.40 and annotation ITAG2.3, tomato accessions, *Solanum pennellii*, *Nicotiana benthamiana* draft genome, pepper genome and *Nicotiana tabacum* genome are available on JBrowse. Eggplant genome will be added in the next months.

To take advantage of the newly available genome sequences, new web tools have been developed at SGN. One example is the SGN Virus-Induced Gene Silencing (VIGS) tool (http://solgenomics.net/tools/vigs), created to help researchers design VIGS constructs (Fernandez-Pozo *et al.*, submitted). This tool was developed to be highly interactive using AJAX (28) for the communications between the front end and the back end, allowing fast calculations without reloading the website. It also uses HTML 5 to draw graphical elements, which allows the reorganization and redrawing of elements on the fly. JQuery (29) (http://jquery.com/) and other Javascript libraries are used to improve the user experience. In recent years, SGN has made use of these web languages and libraries on the new breeding tools and other software, making them more user-friendly and interactive. Another popular tool for SGN users is the tomato unigene converter, which can translate old unigenes names used in publications, such as microarray experiments, to the newest unigene version or the gene name assigned in the reference genome.

## THE SGN PHENOTYPE DATABASE

For genotype to phenotype questions, phenotypic information is of obvious importance and SGN has a suite of tools to deal with phenotypic information. Phenotypes are stored in the Chado Natural Diversity schema (30) that was co-developed by a group of MODs, including SGN and the Genome Database for Rosaceae (31). Each database developed their own user interface around the core database schema. The central concept in the Chado schema is the plant line or stock, which can be characterized with phenotypic data and where the traits are stored using ontologies and other metadata.

There are different types of stocks, including ‘accession’ and ‘plot’. Accessions are plant lines that are available as germplasm, whereas plots are specific accessions that have been planted in the field and which are associated with a trial and other metadata, such as the field location and the field layout. The distinction between accessions and plots is important in a breeding program because selection decisions are made at the level of accessions based on phenotypic data from (sometimes multiple) plots derived from that accession. Information about the experimental design of the field layout (consisting of plots) is necessary to make assessments of an accession from raw plot data. Phenotypes are usually associated with plots to provide all the necessary metadata for the phenotyping experiment. In a phenotyping experiment, the actual phenotypic values are recorded for each plot, but also includes the date of the experiment, the operator, notes and other conditions. For the Solanaceae, a phenotype ontology was developed based on previous large-scale phenotyping experiments (5). The ontology was mapped to the standard Plant Ontology for compatibility with other projects. For each group of species, it is possible to load custom ontologies into the database that describe the specific traits of interest for that species. For example, the SGN system has been applied for cassava in Cassavabase, in which a cassava-specific ontology is used to describe the cassava traits. This ontology was developed by the Crop Ontology Consortium (http://cropontology.org/) (32,33), who have also developed ontologies for a wide variety of other plants. It is recommended to use one of these standard ontologies if they are suitable for a given project.

## THE SGN BREEDING FUNCTIONS

The ultimate promise of plant genomics is the improvement of crops. However, until now, the effect of genomics on crop improvement has lagged. How can genomics achieve this promise? The answer is in the application of new breeding paradigms, such as Genomic Selection (8). Genomic Selection was developed for animal breeding and is now being applied to plants. In a nutshell, Genomic Selection uses phenotypic and genotypic information to correlate genotypes with phenotypes, aiming to predict phenotypes from genotypic information. Since genotyping is now fast, relatively cheap and can be done at the seedling stage, the breeding cycles become cheaper and faster. However, these strategies require robust databases of vast amounts of genotypic and phenotypic information, as well as algorithms and interfaces to perform the model building and breeding value prediction. The SGN system was a good starting point for the implementation of a genomic-based breeding system as it already incorporated comprehensive tools to accommodate phenotypic and genotypic information. The system was applied to a cassava breeding project by creating a separate website called Cassavabase. Detailed functionality of Cassavabase will be described in a separate publication.

## CROSSES, PEDIGREES AND FIELD LAYOUTS

Tracking genotype and phenotype within a breeding program presents a number of challenges for a database because the data are more complicated than a simple genotype-phenotype matrix. Breeding lines can be related to or derived from other lines in complex ways. Phenotypes need to be assessed with multiple replicates and sometimes multiple locations. Because breeding lines are sometimes heterogenous mixtures or segregating populations, the identity and source of material needs to be tracked down to the level of the individual plot or plant. The system captures this information by providing breeders with tools for managing their breeding program and thereby automatically populating the database. Important activities in breeding are crossing lines, tracking pedigrees, designing field trials and collecting data. The system provides functionality for all these use cases. It has an easy-to-use cross-function that allows existing accessions to be crossed with each other, selfed or open pollinated. The function is implemented in a simple dialog window in which the parents and type of cross can be selected and other metadata can be provided, such as number of flowers involved in the cross, operator information and number of seeds obtained. The resulting accessions from a cross can be generated in the database when entering the cross, or added later. The newly generated accessions from a cross will automatically have the correct pedigree information in the database and will appear in the pedigree viewer.

Field layouts can be calculated based on several methods, including complete and incomplete block designs (e.g. augmented). The field layout dialog provides a simple way to create the layouts from a list of accessions. The accessions will be distributed in the layout by creating plot entries linked to the accessions. The plot entries then have metadata associated with them, such as the breeding program, the field layout type and the location and ultimately also the phenotypic scores recorded on the field. The field layouts and trait information can be downloaded to a tablet running the Android Field book application (34) for the collection of phenotypes and collected phenotypes can be uploaded to the database.

As a recurring theme, the breeding process requires the creation and maintenance of lists of different types of items, such as accessions or plots, which are needed for different purposes, for example, field layout generation or crosses. To simplify the management of lists, a list manager was implemented and integrated into the website. Lists can store any number of text elements that can designate, for example, accessions or plots. Each list has a name and also has an assigned type; a list of type accession should only contain valid accession identifiers. Each list type has a validation function, which can check every element in the list and will report elements that are invalid. The list interface is tightly integrated with the tools on the website, with many tools requiring the input of a list. To create lists, users can type the list elements one by one in the list creation dialog, but this can be tedious for long lists. Therefore, a number of tools are available to automatically create larger lists. One such tool is the search wizard, which can select a large number of accessions or plots based on the any combination of trial name, year or location. Also, the results of an accession search can be fed directly into a list.

## FUTURE DIRECTIONS

The SGN code is currently used and will be used as the core for new species-specific breeding databases, such as the previously mentioned CassavaBase. The huge increase of SGN data produced in recent years for experiments like RNA-seq and Genotyping-by-Sequencing (35) is a challenge to store as this can not be properly handled by relational databases. The new SGN applications will use indexed files for fast and easy storage as well as retrieval of these data. As a result, SGN will be a mixed database that combines a traditional relational database with NoSQL methods (36) like indexed files for big data. More features for breeders will be added and a Tomato Expression Atlas for several developmental stages and tissues isolated using laser dissection will be developed (already in progress). In general, SGN tools will be focused on integrating the genomic and phenotypic data with expression data and incorporating new tools useful for researchers and breeders. Specifically, a Genomic Selection tool, already implemented on cassavabase.org will be available on SGN. The tool will enable Genomic Selection by breeders who will be able to build prediction models to estimate genomic estimated breeding values of selection candidates. Data analysis and visualization will be made more interactive using modern web programming libraries such as jQuery.

## SUMMARY AND CONCLUSIONS

Advances in genomics have enabled researchers to more efficiently identify and characterize genes. This has been possible in part because of the MODs that make the genome information easily accessible to researchers. The SGN system is a flexible, community-curated, clade-oriented system that integrates a number of Solanaceae species genomes, including tomato, potato and pepper, with comparative features and graphical tools to support genomics researches. The next step in the genomics advances is to transform breeding. This requires novel systems that combine traditional genomics databases with breeding applications. The SGN platform has been expanded in this direction in recent years. The system has been applied to crops outside of SGN itself and is currently used for cassava and sweet potato.
